# The lactate-to-albumin ratio as a novel predictor for post-stroke epilepsy: insights from a large cohort

**DOI:** 10.1093/braincomms/fcag391

**Published:** 2026-10-15

**Authors:** Dandan Chen, Yuanlin Liu, Yingru Zhou, Qi Yang, Xiao Wu, Yunliang Tang

**Affiliations:** Department of Endocrinology and Metabolism, the First Affiliated Hospital, Jiangxi Medical College, Nanchang University, Nanchang, Jiangxi 330006, China; Jiangxi Clinical Research Center for Endocrine and Metabolic Disease, Nanchang, Jiangxi 330006, China; Jiangxi Branch of National Clinical Research Center for Metabolic Disease, Nanchang University, Nanchang, Jiangxi 330006, China; Queen Mary School, Jiangxi Medical College, Nanchang University, Nanchang, Jiangxi 330006, China; Department of Rehabilitation Medicine, the First Affiliated Hospital, Jiangxi Medical College, Nanchang University, Nanchang 330006, China; Department of Rehabilitation Medicine, the First Affiliated Hospital, Jiangxi Medical College, Nanchang University, Nanchang 330006, China; Department of Neurosurgery, Jiangxi Key Laboratory of Neurological Diseases, the First Affiliated Hospital, Jiangxi Medical College, Nanchang University, Nanchang, Jiangxi 330006, China; Department of Rehabilitation Medicine, the First Affiliated Hospital, Jiangxi Medical College, Nanchang University, Nanchang 330006, China

**Keywords:** lactate-to-albumin ratio, post-stroke epilepsy, ischaemic stroke, biomarker, retrospective cohort

## Abstract

Ischaemic stroke (IS) represents a leading cause of adult mortality and long-term disability globally, and post-stroke epilepsy (PSE) constitutes a severe complication that worsens neurological recovery and reduces quality of life, yet reliable early predictive biomarkers remain insufficient. The serum lactate-to-albumin ratio (LAR, mmol/g), an integrated indicator reflecting hypoxic-ischaemic stress, energy metabolic disturbance, and systemic inflammation, has emerged as a promising prognostic marker but has not been investigated for predicting PSE risk. This multicentre retrospective cohort study enrolled 21 459 acute IS patients from four centres to examine the association between admission LAR and 1-year PSE incidence. Multivariable logistic regression, restricted cubic spline (RCS) analysis, subgroup stratification, and sensitivity testing were applied for statistical evaluation. Overall, 936 patients (4.36%) developed PSE within one year. In the fully adjusted model, each 1-unit increment in LAR was associated with an odds ratio (OR) of 1.13 (95% CI: 1.04–1.22, *P* = 0.003) for PSE. Compared with the lowest LAR quartile (Q1), the highest quartile (Q4) conferred a markedly elevated risk (OR = 12.02, 95% CI: 7.91–18.90, *P* < 0.001), with a significant non-linear dose-response relationship and an inflection point at LAR = 0.06. The association was stronger in patients with atrial fibrillation or cortical stroke lesions, and sensitivity analyses confirmed result robustness. Taken together, these data indicate that serum LAR measured at admission behaves as an independent, non-linear correlate of PSE risk after acute IS. As a readily available biomarker derived from routine serum laboratory tests, LAR may serve as a promising candidate for early risk stratification in IS patients, although prospective studies are warranted to confirm its clinical utility.

## Teaser

Wu *et al*. report that a higher lactate-to-albumin ratio at admission is independently and non-linearly associated with an increased risk of epilepsy after ischaemic stroke. This index, derived from routine blood tests, may serve as a novel tool for early risk stratification in stroke patients.

## Graphical Abstract

**Graphical Abstract:**
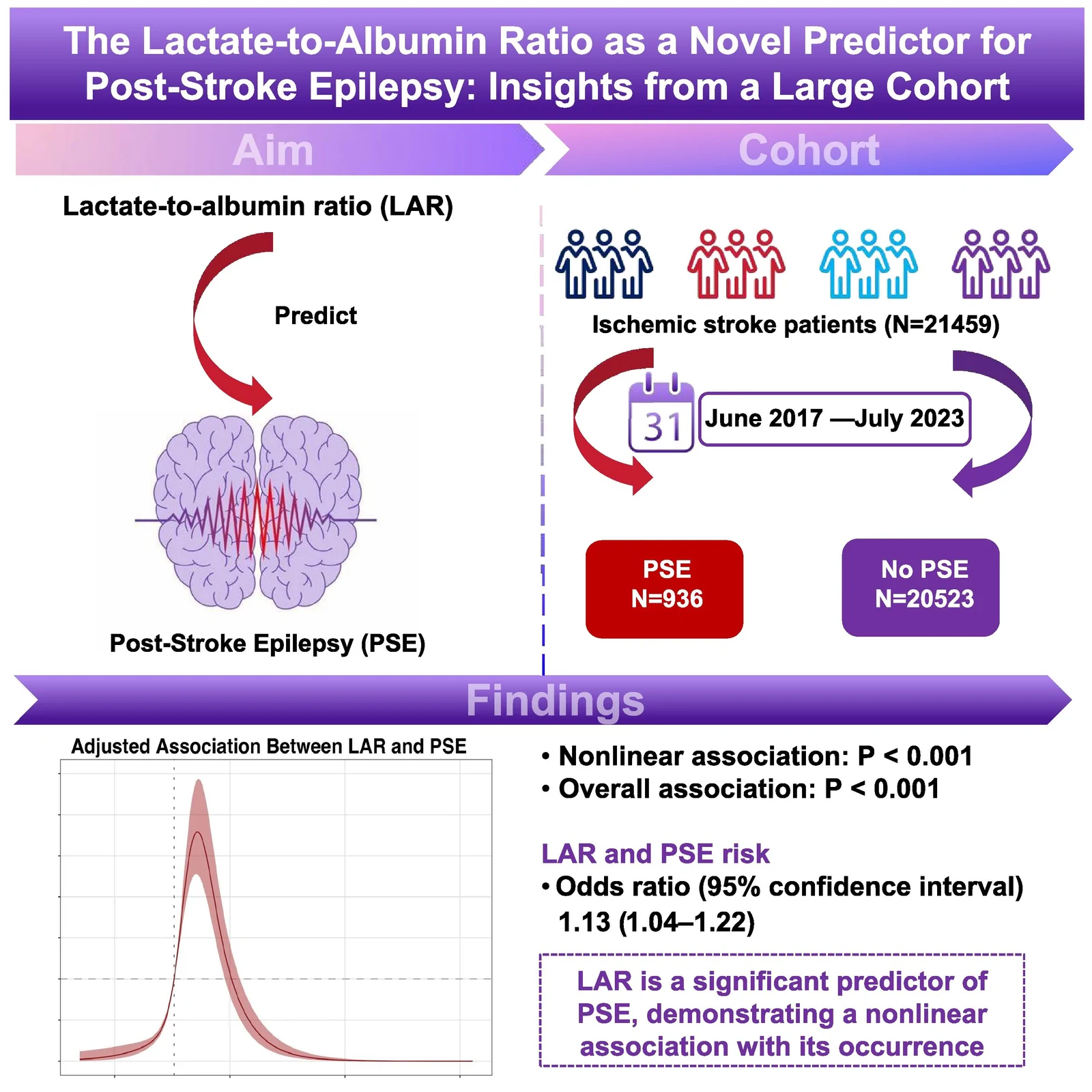

## Introduction

Ischaemic stroke (IS) is a major global health issue and remains one of the top causes of death and long-term disability in adults. It’s responsible for about 5.2% of all deaths worldwide,^1^ which places a heavy social and economic strain on both healthcare systems and affected families. While substantial advancements have been achieved in acute reperfusion therapies and secondary prevention strategies, the management of post-stroke complications remains a critical unmet need that significantly dictates the trajectory of patient rehabilitation.^2,3^ Among these complications, post-stroke epilepsy (PSE) emerges as a particularly debilitating condition that not only exacerbates neurological deficits but also drastically diminishes the quality of life for survivors.^4,5^ The clinical consequences of PSE extend beyond seizures. It raises hospital readmission rates, increases medical costs, and leads to notable declines in functional status.^6,7^ Therefore, identifying accurate early predictors of PSE is essential for the development of personalized management strategies for stroke survivors.

Currently, PSE prediction relies on clinical demographics, stroke severity, electroencephalography, and neuroimaging, yet these markers routinely lack the sensitivity needed for early and precise risk stratification.^8,9^ Established predictors include cortical involvement, higher NIHSS scores, and acute symptomatic seizures,^10,11^ with more recent studies identifying imaging biomarkers such as asymmetric enlarged perivascular spaces^12^ and early epileptiform activity on EEG.^13^ However, the complexity of PSE pathogenesis suggests that single indicators are often inadequate, necessitating integrated biomarkers that reflect both systemic and focal pathological mechanisms.

Among the emerging composite biomarkers, the serum lactate to albumin ratio (LAR) has garnered attention as it integrates hypoxia-ischaemic stress, energy metabolism dysfunction, and systemic inflammation, all of which are closely linked to acute IS (AIS). In recent years, evidence has accumulated that LAR in the prognosis assessment of various diseases.^14-17^ While individual components such as serum lactate, a marker of tissue hypoxia and anaerobic metabolism, and albumin, a key indicator of nutritional status and systemic inflammation, might have been separately linked to severity and prognosis of stroke,^18,19^ their combined utility as a ratio has remained largely unexplored in the realm of PSE prediction. Understanding the LAR-PSE association provides insights into disease mechanisms and enhances early risk stratification. Although no preventive treatments are currently available, identifying high-risk patients enables focused monitoring and informs future research into targeted interventions, with the goal of reducing the long-term burden of PSE.

This retrospective cohort study drew on clinical data from four major hospitals in Chongqing, China, to investigate the association between admission LAR and PSE risk. Using multivariable logistic regression, we analyzed LAR both as a continuous variable and by quartiles to assess its independent association with PSE incidence and to identify potential threshold effects. Our findings may provide a reference for more accurately identifying patients at high risk of PSE, thereby alleviating the burden it places on public health.

## Materials and methods

### Data source and study population

Data for this analysis were sourced from the Dryad Digital Repository, an open-access platform providing freely reusable research data. All contributing authors have waived copyright to these data. This multicentre retrospective study utilized patient records from four hospitals in Chongqing, China. Specifically, data were retrieved from the Chongqing Emergency Centre for the period June 2017 to June 2022, and from Qianjiang Central Hospital, Bishan District People's Hospital, and Yubei District Traditional Chinese Medicine Hospital for the period July 2022 to July 2023. Included patients were aged 18–90 years at admission with a confirmed diagnosis of AIS requiring hospitalization. Exclusion criteria comprised: (i) a history of prior stroke or transient ischaemic attack (TIA); (ii) a history of traumatic brain injury, intracranial tumours, or cerebral vascular malformations potentially predisposing to epilepsy; (iii) a pre-existing epilepsy diagnosis or use of antiseizure medications for any indication; and (iv) mortality within 72 h of stroke onset.

Ethical approval for the present study was not required. This research is a secondary analysis of existing, de-identified, and publicly available data from the Dryad digital repository (https://doi.org/10.5061/dryad.w0vt4b92c).

### Data collection

Clinical and laboratory variables were extracted via a comprehensive review of electronic health records. Collected data encompassed: (i) demographics (age, gender); (ii) comorbidities [uraemia, deep vein thrombosis (DVT), fatty liver disease/steatosis, diabetes mellitus, hypertension, coronary artery disease (CAD), atrial fibrillation, cerebral herniation, hydrocephalus, hyperuricaemia, hyperlipidaemia, hypoproteinemia]; (iii) neuroimaging findings, including involved brain regions (frontal, parietal, temporal, occipital, and insular lobes; basal ganglia; capsula interna; brainstem; rhombencephalon; paraventricular region; centrum semiovale; thalamus; subcortical areas) and vascular territories [anterior cerebral artery (ACA), middle cerebral artery (MCA), posterior cerebral artery (PCA), vertebral artery (VA), basilar artery (BA)]; (iv) cerebral circulation anatomy (anterior circulation, posterior circulation) and plaque location [common carotid artery (CCA), internal carotid artery (ICA), external carotid artery (ECA)]; (v) classification of Large Vessel Disease (based on the TOAST classification); and (vi) extensive laboratory parameters covering haematology [platelet count (PLT), white and red blood cell counts (WBC, RBC)], metabolic markers [glycated haemoglobin (HbA1c), C-reactive protein (CRP), triglycerides (TG), low- and high-density lipoprotein (LDL-c, HDL-c), lactate], liver function [aspartate and alanine aminotransferase (AST, ALT), bilirubin, albumin], renal function [urea, creatinine, uric acid (UA)], coagulation profiles [prothrombin time (PT), activated partial thromboplastin time (APTT), thrombin time (TT), international normalized ratio (INR), D-Dimer, fibrinogen], cardiac enzymes [creatine kinase (CK), CK-MB isoenzyme, lactate dehydrogenase (LDH), hydroxybutyrate dehydrogenase (HBDH)], ischaemia biomarker [ischaemia-modified albumin (IMA)], and serum electrolytes [sodium (Na), potassium (K), chloride (Cl), calcium (Ca), phosphate (P), anion gap (AG), total carbon dioxide].

### Outcome definition

PSE is diagnosed by either two or more unprovoked seizures occurring after the acute phase of stroke (>7 days post-stroke) or, according to updated International League against Epilepsy (ILAE) criteria, a single unprovoked seizure with high recurrence risk.^20^

### Calculation of LAR

The LAR (mmol/g) was calculated by dividing the serum lactate concentration (mmol/L) by the serum albumin concentration (g/L).

### Statistical analysis

Baseline characteristics are presented as mean ± standard deviation (SD) for continuous variables and as frequencies (percentages) for categorical variables. Group comparisons were performed using one-way analysis of variance (ANOVA) or chi-square tests, as appropriate. The association between LAR and PSE was evaluated using multivariable logistic regression. Three models were constructed: Model 1 (unadjusted); Model 2 adjusted for age, gender, NIHSS score, uraemia, DVT, fatty liver, diabetes, hypertension, CAD, atrial fibrillation, and cerebral herniation; and Model 3 additionally adjusted for anterior/posterior circulation involvement, large vessel disease, PLT, WBC, RBC, HbA1c, TG, HDL-c, LDL-c, and creatinine. LAR was analyzed both as a continuous variable and categorized into quartiles. Results are reported as odds ratio (OR) with 95% confidence interval (CI). The dose-response relationship was examined using restricted cubic spline (RCS) analysis with four knots (at the 5th, 35th, 65th, and 95th percentiles), adjusted for Model 3 covariates, and tested for non-linearity. Stratified subgroup analyses were conducted to assess effect modification by baseline and stroke characteristics (DVT, diabetes, hypertension, coronary disease, atrial fibrillation, hydrocephalus, hyperuricaemia, hyperlipidaemia, involved brain regions, involved arteries, gender, carotid plaque locations, subcortical involvement, anterior/posterior circulation). Interaction terms between continuous LAR and each subgroup variable were tested in models adjusted for all other Model 3 covariates (excluding the stratification variable itself). Sensitivity analyses were performed by sequentially excluding patients with cerebral herniation, fatty liver disease/steatosis, or brainstem involvement. All analyses used R software (version 4.5.0), with a two-sided *P*-value < 0.05 considered statistically significant. The R code is available as Supplementary material.

## Results

### Baseline characteristics

Based on the baseline characteristics stratified by LAR quartiles presented in Table S1, several clear trends are observed across the increasing LAR groups (Q1 to Q4). The cohort comprised 21 459 patients with IS, with ∼5375 patients in each quartile. There was a progressive increase in mean age from Q1 (62.60 years) to Q4 (68.35 years). Metabolic and inflammatory markers showed significant gradients: HbA1c, CRP, triglycerides, lactate, and D-dimer levels consistently rose with higher LAR quartiles, while albumin levels declined. The prevalence of comorbidities such as diabetes, hypertension, hypoproteinemia, and DVT markedly increased across quartiles. Notably, the prevalence of PSE related imaging findings, such as involvement of specific arterial territories (e.g. posterior circulation) and conditions like large vessel disease, varied significantly with LAR. Conversely, most neuroanatomical involvement locations (e.g. frontal lobe) and other factors like atrial fibrillation showed less pronounced or non-significant differences. All listed characteristics had a *P*-value < 0.001 except for a few neuroimaging localizations, indicating that a higher LAR is associated with an older population, worse metabolic profiles, heightened systemic inflammation, and a greater burden of specific comorbidities.

### Association of LAR with PSE risk in IS patients

Among the 21 459 patients with IS included in the study, 936 developed PSE. The association between the LAR and PSE incidence is presented in Table 1. Analyzed both as a continuous variable and by quartiles, LAR demonstrated a significant, positive association with PSE risk across all statistical models. Per 1-unit increase in LAR, the adjusted OR was 1.13 (95% CI: 1.04–1.22, *P* = 0.003) in the fully adjusted model (Model 3). When assessed by quartiles, a clear dose-response relationship was evident. Using the first quartile (Q1) as reference, the risk of PSE escalated markedly with higher LAR levels. The adjusted ORs (Model 3) were 3.96 (95% CI: 2.46–6.52) for Q2, 9.57 (95% CI: 6.25–15.16) for Q3, and 12.02 (95% CI: 7.91–18.90) for Q4, all with *P* < 0.001. This graded increase in risk persisted after sequential adjustment for demographics, stroke severity, comorbidities (Model 2), and further for laboratory markers and neuroimaging findings (Model 3), robustly identifying elevated LAR as a strong, independent predictor for the development of PSE.

**Table 1:** Logistic regression analyses of the association between the LAR and PSE incidence in patients with acute ischaemic stroke

Characteristics	Case/total, *n*	Model 1 OR (95% CI)	*P* value	Model 2 OR (95% CI)	*P* value	Model 3 OR (95% CI)	*P* value
LAR (per 1 mmol/g)	936/21 459	1.62 (1.55–1.70)	<0.001	1.74 (1.65–1.84)	<0.001	1.13 (1.04–1.22)	0.003
LAR quartile							
Quartile 1	34/5375	Reference		Reference		Reference	
Quartile 2	59/5383	1.74 (1.15–2.68)	0.010	2.48 (1.62–3.84)	<0.001	3.96 (2.46–6.52)	<0.001
Quartile 3	179/5348	5.44 (3.82–7.99)	<0.001	8.57 (5.96–12.69)	<0.001	9.57 (6.25–15.16)	<0.001
Quartile 4	664/5353	22.24 (15.99–32.06)	<0.001	35.22 (25.01–51.29)	<0.001	12.02 (7.91–18.90)	<0.001

Model 1: unadjusted for any covariates.

Model 2: adjusted for age, gender, NIHSS, uraemia, DVT, fatty liver, diabetes, hypertension, CAD, atrial fibrillation, and cerebral herniation.

Model 3: Model 2 + further adjusted for anterior circulation, posterior circle, large vessel disease, PLT, WBC, RBC, HbA1c, TG, HDL-c, LDL-c, creatinine.

### Non-linear association analysis

RCS regression with multivariable adjustment was utilized to characterize the dose–response relationships between the LAR and the prevalence of PSE across five distinct subgroups: the total study population, the non-large vessel disease subgroup, the large vessel disease subgroup, the NIHSS score 5–14 subgroup, and the NIHSS score >14 subgroup. In the overall study population, an inflection point was detected at an LAR value of 0.06. Figure 1 illustrates the adjusted non-linear association between LAR and PSE prevalence, which was derived from RCS regression incorporating full covariate adjustment. With the median LAR value serving as the reference, both a significant non-linear relationship (*P* for non-linearity < 0.001) and an overall significant association (*P* for overall < 0.001) were observed. Within the non-large vessel disease subgroup, Fig. 2A demonstrates a significant non-linear correlation between LAR and PSE risk among IS participants (*P* < 0.001; *P* for non-linearity < 0.001). Correspondingly, in the large vessel disease subgroup, Fig. 2B reveals a similarly significant non-linear association (*P* < 0.001; *P* for non-linearity < 0.001). For the NIHSS score 5–14 subgroup, Fig. 3A shows a significant non-linear relationship between LAR and PSE risk in IS participants (*P* < 0.001; *P* for non-linearity < 0.001). Likewise, in the NIHSS score >14 subgroup, Fig. 3B indicates a significant non-linear association (*P* < 0.001; *P* for non-linearity < 0.001).

**Figure 1:**
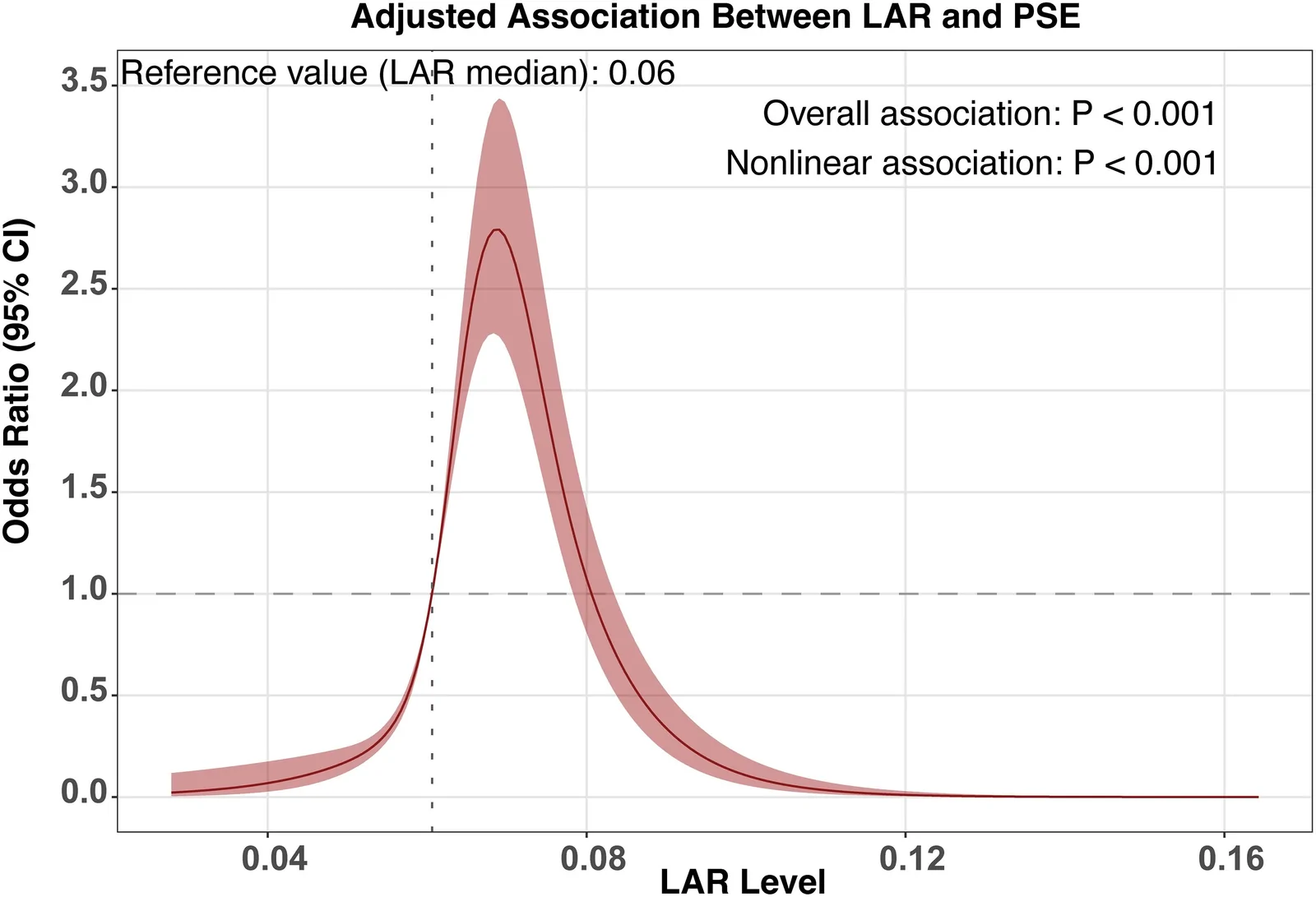
**Dose-response relationship between LAR (lactate-to-albumin ratio) and the risk of PSE (post-stroke epilepsy) in the entire cohort.** Multivariable-adjusted RCS regression showed a non-linear association between LAR and PSE occurrence (*n* = 21 459). Adjusted for age, gender, NIHSS (National Institutes of Health Stroke Scale), uraemia, DVT, fatty liver, diabetes, hypertension, CAD, atrial fibrillation, and cerebral herniation, anterior circulation, posterior circulation, large vessel disease, PLT (platelet count), WBC (white blood cell count), RBC (red blood cell count), HbA1c (glycated haemoglobin A1c), TG (triglycerides), HDL-c (high-density lipoprotein cholesterol), LDL-c (low-density lipoprotein cholesterol), and creatinine. Statistical methods: Multivariable logistic regression with a RCS function for LAR (4 knots placed at the 5th, 35th, 65th, and 95th percentiles of the distribution) was fitted to evaluate the adjusted dose-response relationship between LAR and PSE.

**Figure 2:**
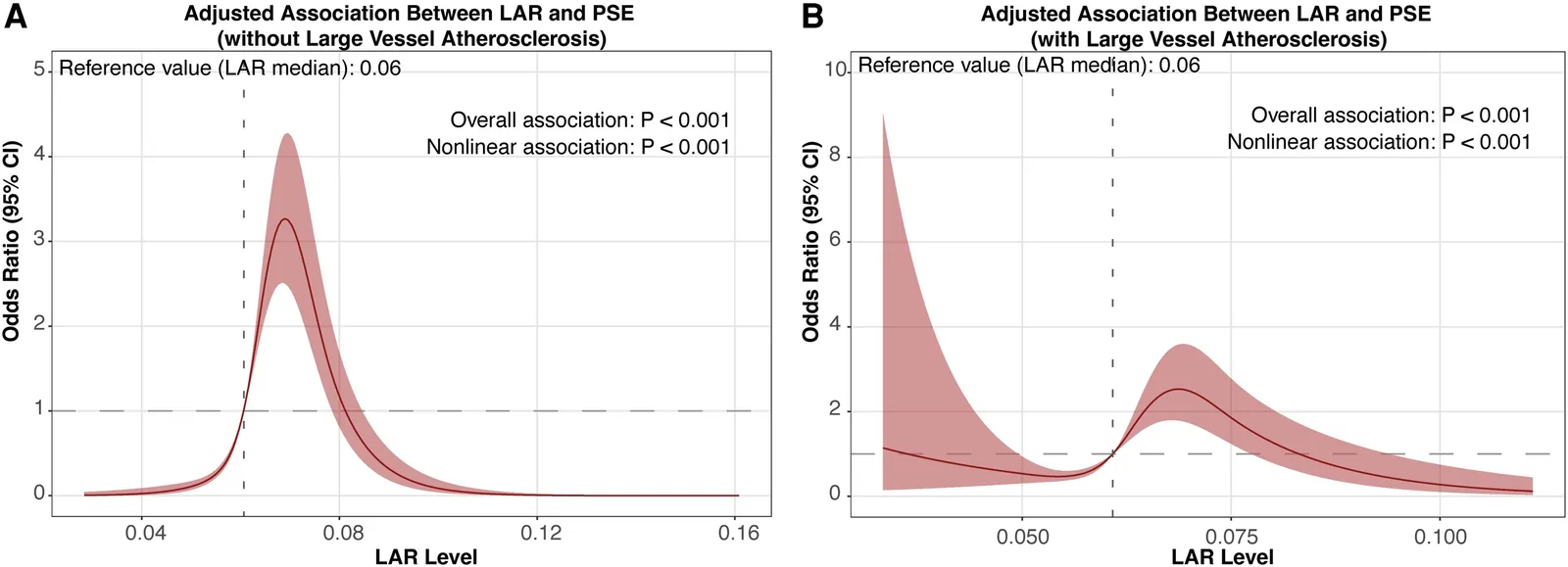
**RCS analysis of the association between LAR (lactate-to-albumin ratio) and PSE (post-stroke epilepsy) incidence in ischaemic stroke patients, stratified by large vessel disease.** (**A**) Without the large vessel disease group (*n* = 15 954) and (**B**) with large vessel disease group (*n* = 5505). Adjusted for age, gender, NIHSS (National Institutes of Health Stroke Scale), uraemia, DVT, fatty liver, diabetes, hypertension, CAD, atrial fibrillation, and cerebral herniation, anterior circulation, posterior circulation, PLT (platelet count), WBC (white blood cell count), RBC (red blood cell count), HbA1c (glycated haemoglobin A1c), TG (triglycerides), HDL-c (high-density lipoprotein cholesterol), LDL-c (low-density lipoprotein cholesterol), and creatinine. Statistical methods: Multivariable logistic regression with a RCS function for LAR (4 knots placed at the 5th, 35th, 65th, and 95th percentiles of the distribution) was fitted to evaluate the adjusted dose-response relationship between LAR and PSE.

**Figure 3:**
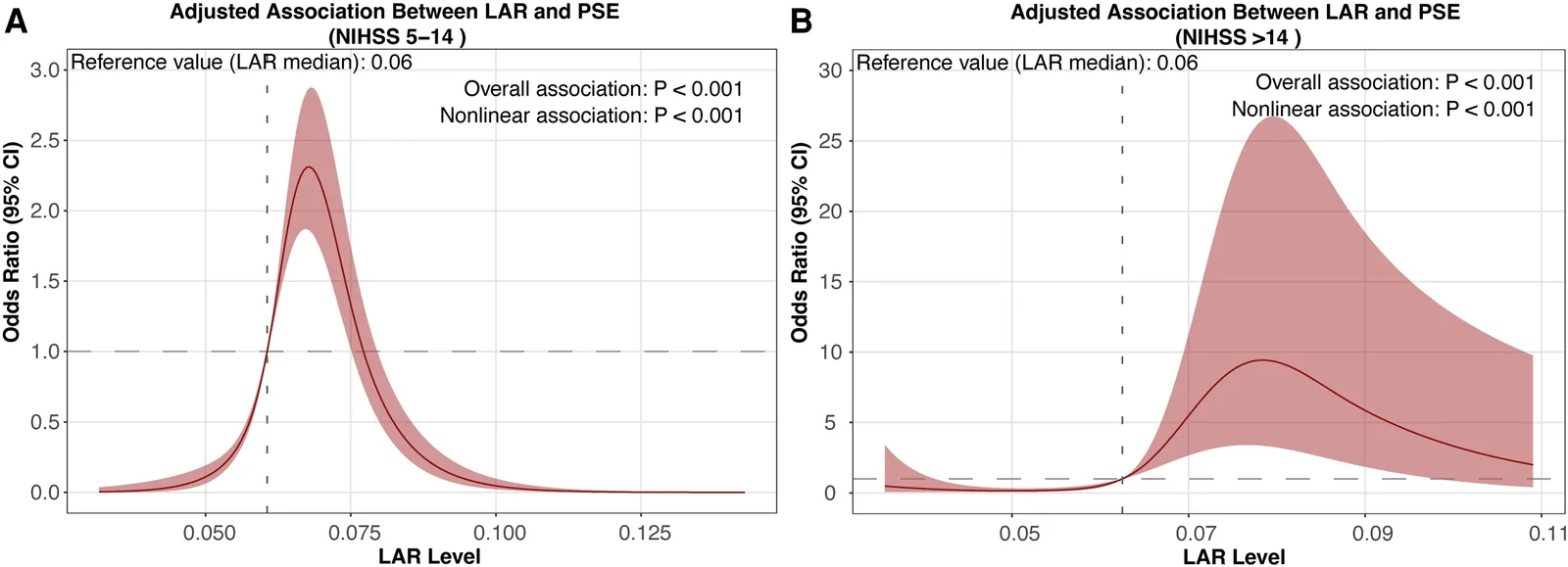
**RCS analysis of LAR (lactate-to-albumin ratio) and PSE (post-stroke epilepsy) incidence in ischaemic stroke patients, stratified by NIHSS (National Institutes of Health Stroke Scale) score.** (**A**) NIHSS (5–14) group (*n* = 19 371) and (**B**) NIHSS (>14) group (*n* = 559). Adjusted for age, gender, uraemia, DVT, fatty liver, diabetes, hypertension, CAD, atrial fibrillation, and cerebral herniation, anterior circulation, posterior circulation, PLT (platelet count), WBC (white blood cell count), RBC (red blood cell count), HbA1c (glycated haemoglobin A1c), TG (triglycerides), HDL-c (high-density lipoprotein cholesterol), LDL-c (low-density lipoprotein cholesterol), and creatinine. Statistical methods: Multivariable logistic regression with a RCS function for LAR (4 knots placed at the 5th, 35th, 65th, and 95th percentiles of the distribution) was fitted to evaluate the adjusted dose-response relationship between LAR and PSE.

### Subgroup analyses

Subgroup analyses were performed to assess the potential effect modification of various baseline and stroke-related factors on the association between the LAR and PSE (Table S2). The positive association was significantly stronger in several key patient subgroups. Notably, the presence of certain comorbidities substantially amplified the risk: the OR for the LAR-PSE association was 1.41 (95% CI 1.05–1.90, *P* = 0.022) in patients with DVT, 1.25 (1.08–1.44, *P* = 0.002) in those with coronary disease, and 1.47 (1.18–1.81, *P* < 0.001) in patients with atrial fibrillation. Conversely, the association was markedly attenuated and non-significant in patients with diabetes (OR = 1.00, 95% CI: 0.89–1.12, *P* = 0.972) or hypertension (OR = 1.02, 95% CI: 0.94–1.11, *P* = 0.588), with significant interaction *P*-values of <0.001 for both. The anatomical location of the stroke also served as an important effect modifier. A significantly stronger association was observed when the stroke involved the parietal lobe (OR = 1.71, 95% CI: 1.28–2.30, *P* < 0.001), temporal lobe (OR = 2.08, 95% CI: 1.48–3.17, *P* < 0.001), occipital lobe (OR = 5.38, 95% CI: 1.95–20.88, *P* = 0.004), basal ganglia (OR = 2.23, 95% CI: 1.57–3.26, *P* < 0.001), or the paraventricular region (OR = 2.94, 95% CI: 1.92–4.57, *P* < 0.001). Furthermore, the presence of external carotid artery plaque was linked to an exceptionally high point estimate (OR = 10.45, 95% CI: 1.74–135.57, *P* = 0.030). No significant interaction was found for factors such as gender, hydrocephalus, or stroke in the anterior versus posterior circulation, indicating that the LAR-PSE association was consistent across these subgroups. All models were adjusted for a comprehensive set of covariates.

### Sensitivity analyses

To ensure the robustness of our primary findings, we performed a series of sensitivity analyses. The positive association between the LAR and PSE risk remained consistently significant across multiple, sequentially restricted patient cohorts. In the subgroup of patients aged ≤80 years, the fully adjusted Model 3 demonstrated a significant association (OR = 1.11, 95% CI: 1.02–1.21, *P* = 0.02) (Table S3). This association persisted after excluding patients with cerebral herniation (OR = 1.13, 95% CI: 1.05–1.22, *P* = 0.002) (Table S4). Furthermore, to mitigate potential confounding from metabolic comorbidities and specific stroke localizations, we repeated the analysis after excluding patients with fatty liver disease or hepatic steatosis, which yielded a similar result (OR = 1.12, 95% CI: 1.03–1.22, *P* = 0.01) (Table S5). Finally, upon excluding patients with brainstem involvement, the LAR remained a significant predictor of PSE (OR = 1.13, 95% CI: 1.04–1.22, *P* = 0.003) (Table S6). Critically, in all these sensitivity analyses, the graded increase in PSE risk across ascending LAR quartiles was consistently observed and remained highly statistically significant, robustly confirming the stability and reliability of our main results.

## Discussion

In the current study, we systematically evaluated the prognostic value of the LAR, a readily accessible index reflecting hypoxia-ischaemic stress, energy metabolism disorder, and systemic inflammation, in predicting PSE risk among AIS survivors. By leveraging a large-scale cohort, we demonstrated that admission LAR levels are independently associated with risk of PSE, exhibiting a robust non-linear dose-response relationship. Collectively, these observations support the use of LAR for identifying patients who warrant closer surveillance, which may in turn reduce the overall burden of PSE.

Recently, numerous risk factors have been identified for PSE.^6,21,22^ However, despite the availability of these predictive tools, these traditional markers often lack the sensitivity to achieve early, precise risk stratification, and the complexity of the underlying mechanisms continues to pose a significant challenge for the accurate clinical identification of these patients. Our findings establish LAR as a practical and potent biomarker, potentially guiding targeted monitoring for different IS patients.

Our study provides the first systematic elucidation of the relationship between LAR and PSE. However, the specific mechanisms underlying LAR and PSE remain unclear. LAR might serve as a potential biomarker associated with the development of PSE, likely mediated through interconnected molecular and physiological mechanisms. Elevated lactate levels signal a shift to anaerobic metabolism, often resulting from cerebral hypoxia or ischaemia following a stroke.^23,24^ This build-up of lactate can induce intracellular acidosis, which impairs ion channel function, especially those involved in glutamate release and GABAergic inhibition, ultimately lowering the seizure threshold and increasing neuronal hyperexcitability.^25-27^ At the same time, reduced serum albumin may reflect systemic inflammation, blood-brain barrier disruption, or oxidative stress.^28-30^ Albumin possesses inherent antioxidant and anti-inflammatory properties, so its decline could diminish endogenous neuroprotection. This may heighten BBB permeability, allowing pro-epileptogenic agents such as iron and cytokines to enter the brain parenchyma.^31,32^ Moreover, the combination of high lactate and low albumin might worsen neuroinflammation. Lactate can trigger inflammatory pathways, including NF-κB and NLRP3 inflammasome signalling, leading to the release of interleukin-1β and other cytokines that promote epileptogenesis.^33-35^ Albumin deficiency, on the other hand, may hinder the clearance of toxic metabolites and reduce the capacity to buffer oxidative damage, leaving neurons more susceptible to excitotoxicity.^28^ Additionally, elevated lactate points to impaired mitochondrial function and energy failure, which can disrupt ATP-dependent processes. This affects synaptic plasticity and encourages hyper-synchronization within neural networks.^36,37^ In summary, LAR appears to reflect an integrative disturbance involving metabolic dysfunction, neuroinflammation, and compromised neurovascular integrity-together creating a microenvironment that supports the development of PSE.

Large vessel disease is closely related to stroke severity. Occlusions in arteries such as the middle cerebral artery is a significant risk factor for PSE because they frequently lead to larger, more severe cortical infarcts.^38^ These extensive infarctions create a greater burden of neuronal injury and gliosis, which serve as key substrates for the development of epileptic foci. In the present study, we also performed RCS analysis to evaluate the association between LAR and PSE in individuals with or without large vessel disease. It was found that LAR and PSE exhibited a non-linear association both in populations with and without large vessel disease, which was consistent with the results from the entire cohort. In addition, stroke severity is often associated with the risk of PSE. We also employed RCS analysis to examine the relationship between LAR and PSE across populations with different NIHSS scores. Our results demonstrated that LAR had a non-linear association with PSE in the studied individuals, with NIHSS (5–14) or NIHSS > 14. This relationship mirrored the results observed in the entire cohort. Based on the above results, it can be inferred that our findings are reliable and robust.

Subgroup analyses indicated that the strength of the LAR-PSE relationship was significantly modified by specific patient characteristics: it was markedly stronger in individuals with comorbidities such as DVT, CAD, or atrial fibrillation, whereas it was attenuated and became non-significant in those with diabetes or hypertension. The most likely explanation is insufficient statistical power due to the low incidence of PSE in the overall population and the relatively small number of patients with comorbidities such as DVT, CAD, or atrial fibrillation. Moreover, stroke localization served as an important effect modifier, with a significantly stronger association observed when infarctions involved cortical regions (parietal, temporal, or occipital lobes) or subcortical structures (basal ganglia or paraventricular region). In contrast, factors such as gender and anterior versus posterior circulation stroke location did not significantly modify the association, indicating consistency across these subgroups. The robustness of the primary finding was further reinforced by sensitivity analyses, in which the positive association and clear dose-response relationship across LAR quartiles persisted after sequentially excluding patients with age > 80 years, cerebral herniation, fatty liver disease/steatosis, or brainstem involvement, confirming that LAR’s predictive value is independent of these specific conditions. These results confirm LAR as a novel predictor, demonstrate its utility for risk stratification in patients with specific comorbidities and stroke localizations, and support the generalizability of its prognostic value in the studied AIS population.

The present work has several notable strengths. First, it draws on a multicentre cohort of more than 21 000 patients with IS, which affords substantial statistical power. Second, stringent multivariable adjustment together with sensitivity testing corroborated a stable, independent and non-linear LAR-PSE relationship. Third, LAR can be obtained from standard blood chemistry, making it a convenient and inexpensive adjunct for early risk stratification with clear translational appeal.

Several limitations of this study warrant consideration. Firstly, the retrospective design and reliance on electronic health records from four hospitals in a single region of China with different enrollment periods, which may introduce confounding bias. Secondly, though we have adjusted for many potential confounders, the lack analysis of some important confounders (such as intravenous alteplase, endovascular therapy, and smoking) may introduce bias. Thirdly, although the overall cohort was large, the small sample sizes in some subgroups likely resulted in insufficient statistical power. This may have limited our ability to detect significant associations or led to unstable risk estimates within those specific strata, highlighting a need for caution when interpreting the subgroup analyses. Fourthly, the calculation of LAR from single-point, early-phase laboratory measurements may not fully capture the dynamic interplay between lactate metabolism and nutritional status over the critical post-stroke period, potentially oversimplifying a complex biological relationship. Fifthly, the follow-up period of this study was one year after stroke onset, while many patients may have developed PSE by that time, research suggests that the process can take longer,^39,40^ implying that the prevalence of PSE may have been underestimated.

In summary, this large cohort shows that LAR at admission is independently and non-linearly related to the risk of PSE following AIS. As a readily available metric from routine blood tests, LAR offers a novel, practical tool for early risk stratification. Validation in prospective settings will be required before its predictive value and clinical implications can be established.

## Supplementary Material

fcag391_Supplementary_Data

## Data Availability

All the data and materials that are required to reproduce these findings can be shared by contacting the corresponding author. The R code is available as Supplementary material.
