# Supplementary material for: The lactate-to-albumin ratio as a novel predictor for post-stroke epilepsy: insights from a large cohort
Source: Brain Commun. 2026 Oct 15;8(5):fcag391. doi: 10.1093/braincomms/fcag391 (PMC13711666; doi:10.1093/braincomms/fcag391)
Supplement: Supplementary file 1 [file fcag391_supplementary_data.zip › Supplementary_material.docx]

| **Table S1. Baseline characteristics stratified by LAR quartiles.** | | | | | | |
| --- | --- | --- | --- | --- | --- | --- |
| **Characteristic** |  | **Q1 (n=5375)** | **Q2 (n=5383)** | **Q3 (n=5348)** | **Q4 (n=5353)** | **P value** |
| Age, years |  | 62.60 (14.08) | 66.42 (11.53) | 68.30 (10.99) | 68.35 (11.66) | <0.001 |
| PLT, ×10^9/L (cells/L) |  | 187.80 (31.38) | 191.99 (24.18) | 191.69 (24.44) | 188.12 (27.18) | <0.001 |
| WBC, ×10^9/L (cells/L) |  | 8.15 (1.40) | 8.08 (1.05) | 8.44 (1.21) | 9.23 (2.17) | <0.001 |
| RBC, ×10^12/L (cells/L) |  | 4.38 (0.33) | 4.31 (0.29) | 4.29 (0.31) | 4.27 (0.34) | <0.001 |
| HbA1c, % |  | 6.28 (0.76) | 6.55 (0.74) | 6.77 (0.89) | 7.08 (1.08) | <0.001 |
| CRP, mg/L |  | 11.45 (17.84) | 14.12 (19.01) | 15.34 (16.24) | 27.25 (31.05) | <0.001 |
| TG, mmol/L |  | 1.39 (0.40) | 1.54 (0.38) | 1.59 (0.38) | 1.66 (0.52) | <0.001 |
| LDL-c, mmol/L |  | 2.61 (0.37) | 2.71 (0.29) | 2.71 (0.32) | 2.72 (0.44) | <0.001 |
| HDL-c, mmol/L |  | 1.27 (0.15) | 1.26 (0.14) | 1.25 (0.15) | 1.22 (0.15) | <0.001 |
| AST, U/L |  | 25.44 (11.36) | 26.05 (15.55) | 26.20 (12.77) | 28.70 (13.33) | <0.001 |
| ALT, U/L |  | 22.71 (9.02) | 23.81 (8.94) | 24.81 (10.52) | 26.20 (11.57) | <0.001 |
| Bilirubin, µmol/L |  | 15.88 (4.02) | 14.72 (3.34) | 15.30 (4.31) | 15.15 (4.20) | <0.001 |
| Albumin, g/L |  | 41.59 (1.68) | 41.18 (1.80) | 40.81 (2.15) | 40.07 (2.87) | <0.001 |
| Urea, mmol/L |  | 5.81 (1.34) | 6.22 (0.99) | 6.62 (1.09) | 6.96 (1.87) | <0.001 |
| Creatinine, µmol/L |  | 79.29 (44.94) | 79.77 (40.01) | 84.50 (40.77) | 96.03 (70.82) | <0.001 |
| UA, µmol/L |  | 340.23 (58.79) | 340.77 (55.66) | 344.73 (54.59) | 350.87 (65.18) | <0.001 |
| PT, seconds |  | 13.68 (0.70) | 13.68 (0.71) | 13.83 (0.98) | 14.18 (1.75) | <0.001 |
| APTT, seconds |  | 35.76 (2.18) | 35.36 (1.92) | 35.51 (2.10) | 35.99 (2.84) | <0.001 |
| TT, seconds |  | 16.30 (0.72) | 16.43 (0.49) | 16.54 (0.54) | 16.50 (0.65) | <0.001 |
| INR, ratio |  | 1.06 (0.10) | 1.06 (0.10) | 1.08 (0.13) | 1.12 (0.22) | <0.001 |
| D-dimer, ng/mL |  | 0.96 (0.57) | 1.08 (0.84) | 1.20 (1.08) | 2.05 (2.36) | <0.001 |
| Fibrinogen, g/L |  | 3.46 (0.48) | 3.56 (0.37) | 3.62 (0.42) | 3.74 (0.57) | <0.001 |
| CK, U/L |  | 189.64 (173.66) | 171.73 (192.20) | 190.03 (213.81) | 376.16 (629.39) | <0.001 |
| CK-MB, U/L |  | 13.79 (7.03) | 14.35 (10.83) | 14.42 (10.04) | 17.47 (13.89) | <0.001 |
| LDH, U/L |  | 213.20 (100.45) | 217.38 (78.80) | 213.42 (52.81) | 231.31 (75.16) | <0.001 |
| HBDH, U/L |  | 171.79 (40.14) | 176.36 (54.53) | 174.70 (42.35) | 186.14 (56.51) | <0.001 |
| IMA, U/mL |  | 76.62 (18.55) | 75.04 (12.14) | 74.55 (8.91) | 76.50 (9.27) | <0.001 |
| Na, mmol/L |  | 138.60 (1.94) | 138.87 (1.47) | 138.60 (1.60) | 138.21 (1.91) | <0.001 |
| K, mmol/L |  | 3.77 (0.18) | 3.79 (0.14) | 3.81 (0.15) | 3.78 (0.20) | <0.001 |
| Cl, mmol/L |  | 103.57 (2.17) | 103.83 (1.55) | 103.41 (1.78) | 102.65 (2.12) | <0.001 |
| Ca, mmol/L |  | 2.21 (0.07) | 2.21 (0.06) | 2.20 (0.06) | 2.20 (0.08) | <0.001 |
| P, mmol/L |  | 1.00 (0.10) | 0.99 (0.08) | 1.00 (0.08) | 0.98 (0.11) | <0.001 |
| Lactate, mmol/L |  | 2.12 (0.19) | 2.39 (0.11) | 2.59 (0.15) | 2.99 (0.47) | <0.001 |
| AG, mmol/L |  | 12.17 (1.50) | 12.16 (1.11) | 12.34 (1.26) | 12.85 (1.47) | <0.001 |
| Total carbon dioxide, mmol/L |  | 22.82 (1.35) | 22.86 (1.00) | 22.84 (1.07) | 22.62 (1.39) | <0.001 |
| Uremia (%) | No | 5315 (98.9) | 5356 (99.5) | 5328 (99.6) | 5270 (98.4) | <0.001 |
|  | Yes | 60 (1.1) | 27 (0.5) | 20 (0.4) | 83 (1.6) |  |
| DVT (%) | No | 5252 (97.7) | 5160 (95.9) | 5034 (94.1) | 4689 (87.6) | <0.001 |
|  | Yes | 123 (2.3) | 223 (4.1) | 314 (5.9) | 664 (12.4) |  |
| Fatty liver disease or steatosis (%) | No | 4699 (87.4) | 4222 (78.4) | 4014 (75.1) | 4279 (79.9) | <0.001 |
|  | Yes | 676 (12.6) | 1161 (21.6) | 1334 (24.9) | 1074 (20.1) |  |
| Diabetes (%) | No | 4517 (84.0) | 3911 (72.7) | 3203 (59.9) | 2506 (46.8) | <0.001 |
|  | Yes | 858 (16.0) | 1472 (27.3) | 2145 (40.1) | 2847 (53.2) |  |
| Hypertension (%) | No | 2233 (41.5) | 1801 (33.5) | 1463 (27.4) | 1211 (22.6) | <0.001 |
|  | Yes | 3142 (58.5) | 3582 (66.5) | 3885 (72.6) | 4142 (77.4) |  |
| CAD (%) | No | 3220 (59.9) | 2885 (53.6) | 2769 (51.8) | 2913 (54.4) | <0.001 |
|  | Yes | 2155 (40.1) | 2498 (46.4) | 2579 (48.2) | 2440 (45.6) |  |
| Atrial fibrillation (%) | No | 4891 (91.0) | 4945 (91.9) | 4835 (90.4) | 4745 (88.6) | <0.001 |
|  | Yes | 484 (9.0) | 438 (8.1) | 513 (9.6) | 608 (11.4) |  |
| Cerebral herniation (%) | No | 5338 (99.3) | 5363 (99.6) | 5300 (99.1) | 5277 (98.6) | <0.001 |
|  | Yes | 37 (0.7) | 20 (0.4) | 48 (0.9) | 76 (1.4) |  |
| Hydrocephalus (%) | No | 5301 (98.6) | 5336 (99.1) | 5298 (99.1) | 5243 (97.9) | <0.001 |
|  | Yes | 74 (1.4) | 47 (0.9) | 50 (0.9) | 110 (2.1) |  |
| Hyperuricemia (%) | No | 4963 (92.3) | 4889 (90.8) | 4664 (87.2) | 4596 (85.9) | <0.001 |
|  | Yes | 412 (7.7) | 494 (9.2) | 684 (12.8) | 757 (14.1) |  |
| Hyperlipidemia (%) | No | 4438 (82.6) | 4075 (75.7) | 4041 (75.6) | 4436 (82.9) | <0.001 |
|  | Yes | 937 (17.4) | 1308 (24.3) | 1307 (24.4) | 917 (17.1) |  |
| Hypoproteinemia (%) | No | 5285 (98.3) | 5113 (95.0) | 4721 (88.3) | 3919 (73.2) | <0.001 |
|  | Yes | 90 (1.7) | 270 (5.0) | 627 (11.7) | 1434 (26.8) |  |
| Frontal lobe (%) | No | 5134 (95.5) | 5129 (95.3) | 5157 (96.4) | 5120 (95.6) | 0.021 |
|  | Yes | 241 (4.5) | 254 (4.7) | 191 (3.6) | 233 (4.4) |  |
| Parietal lobe (%) | No | 5190 (96.6) | 5201 (96.6) | 5215 (97.5) | 5190 (97.0) | 0.016 |
|  | Yes | 185 (3.4) | 182 (3.4) | 133 (2.5) | 163 (3.0) |  |
| Temporal lobe (%) | No | 5181 (96.4) | 5229 (97.1) | 5218 (97.6) | 5195 (97.0) | 0.004 |
|  | Yes | 194 (3.6) | 154 (2.9) | 130 (2.4) | 158 (3.0) |  |
| Occipital lobe (%) | No | 5261 (97.9) | 5290 (98.3) | 5260 (98.4) | 5253 (98.1) | 0.276 |
|  | Yes | 114 (2.1) | 93 (1.7) | 88 (1.6) | 100 (1.9) |  |
| Insular lobe (%) | No | 5307 (98.7) | 5298 (98.4) | 5285 (98.8) | 5290 (98.8) | 0.206 |
|  | Yes | 68 (1.3) | 85 (1.6) | 63 (1.2) | 63 (1.2) |  |
| Basal ganglia (%) | No | 5059 (94.1) | 5137 (95.4) | 5144 (96.2) | 5151 (96.2) | <0.001 |
|  | Yes | 316 (5.9) | 246 (4.6) | 204 (3.8) | 202 (3.8) |  |
| Capsula interna (%) | No | 5373 (100.0) | 5379 (99.9) | 5342 (99.9) | 5351 (100.0) | 0.367 |
|  | Yes | 2 (0.0) | 4 (0.1) | 6 (0.1) | 2 (0.0) |  |
| Brainstem (%) | No | 5312 (98.8) | 5305 (98.6) | 5281 (98.7) | 5289 (98.8) | 0.564 |
|  | Yes | 63 (1.2) | 78 (1.4) | 67 (1.3) | 64 (1.2) |  |
| Rhombencephalon (%) | No | 5276 (98.2) | 5281 (98.1) | 5238 (97.9) | 5222 (97.6) | 0.113 |
|  | Yes | 99 (1.8) | 102 (1.9) | 110 (2.1) | 131 (2.4) |  |
| Paraventricular (%) | No | 5089 (94.7) | 5030 (93.4) | 5131 (95.9) | 5162 (96.4) | <0.001 |
|  | Yes | 286 (5.3) | 353 (6.6) | 217 (4.1) | 191 (3.6) |  |
| Centrum semiovale (%) | No | 5208 (96.9) | 5149 (95.7) | 5229 (97.8) | 5263 (98.3) | <0.001 |
|  | Yes | 167 (3.1) | 234 (4.3) | 119 (2.2) | 90 (1.7) |  |
| Thalamus (%) | No | 5302 (98.6) | 5316 (98.8) | 5289 (98.9) | 5315 (99.3) | 0.008 |
|  | Yes | 73 (1.4) | 67 (1.2) | 59 (1.1) | 38 (0.7) |  |
| Anterior cerebral artery (%) | No | 5248 (97.6) | 5309 (98.6) | 5313 (99.3) | 5315 (99.3) | <0.001 |
|  | Yes | 127 (2.4) | 74 (1.4) | 35 (0.7) | 38 (0.7) |  |
| Middle cerebral artery (%) | No | 5076 (94.4) | 5193 (96.5) | 5198 (97.2) | 5164 (96.5) | <0.001 |
|  | Yes | 299 (5.6) | 190 (3.5) | 150 (2.8) | 189 (3.5) |  |
| Posterior cerebral artery (%) | No | 5367 (99.9) | 5360 (99.6) | 5330 (99.7) | 5340 (99.8) | 0.045 |
|  | Yes | 8 (0.1) | 23 (0.4) | 18 (0.3) | 13 (0.2) |  |
| Vertebral artery (%) | No | 5166 (96.1) | 5223 (97.0) | 5221 (97.6) | 5197 (97.1) | <0.001 |
|  | Yes | 209 (3.9) | 160 (3.0) | 127 (2.4) | 156 (2.9) |  |
| Basilar artery (%) | No | 5308 (98.8) | 5330 (99.0) | 5316 (99.4) | 5314 (99.3) | 0.002 |
|  | Yes | 67 (1.2) | 53 (1.0) | 32 (0.6) | 39 (0.7) |  |
| CCA plaque (%) | No | 4460 (83.0) | 4037 (75.0) | 3859 (72.2) | 4280 (80.0) | <0.001 |
|  | Yes | 915 (17.0) | 1346 (25.0) | 1489 (27.8) | 1073 (20.0) |  |
| ICA plaque (%) | No | 4950 (92.1) | 5043 (93.7) | 4981 (93.1) | 5023 (93.8) | 0.001 |
|  | Yes | 425 (7.9) | 340 (6.3) | 367 (6.9) | 330 (6.2) |  |
| ECA plaque (%) | No | 5335 (99.3) | 5328 (99.0) | 5295 (99.0) | 5300 (99.0) | 0.407 |
|  | Yes | 40 (0.7) | 55 (1.0) | 53 (1.0) | 53 (1.0) |  |
| Subcortical areas (%) | No | 4728 (88.0) | 4670 (86.8) | 4789 (89.5) | 4829 (90.2) | <0.001 |
|  | Yes | 647 (12.0) | 713 (13.2) | 559 (10.5) | 524 (9.8) |  |
| Anterior circulation (%) | No | 4877 (90.7) | 5043 (93.7) | 5064 (94.7) | 5062 (94.6) | <0.001 |
|  | Yes | 498 (9.3) | 340 (6.3) | 284 (5.3) | 291 (5.4) |  |
| Posterior circle (%) | No | 3982 (74.1) | 4068 (75.6) | 4255 (79.6) | 4653 (86.9) | <0.001 |
|  | Yes | 1393 (25.9) | 1315 (24.4) | 1093 (20.4) | 700 (13.1) |  |
| Large vessel disease (%) | No | 4191 (78.0) | 3874 (72.0) | 3728 (69.7) | 4161 (77.7) | <0.001 |
|  | Yes | 1184 (22.0) | 1509 (28.0) | 1620 (30.3) | 1192 (22.3) |  |
| NIHSS (%) | <5 | 394 (7.3) | 419 (7.8) | 371 (6.9) | 345 (6.4) | <0.001 |
|  | 5-14 | 4821 (89.7) | 4864 (90.4) | 4874 (91.1) | 4812 (89.9) |  |
|  | >14 | 160 (3.0) | 100 (1.9) | 103 (1.9) | 196 (3.7) |  |

| **Table S2. Subgroup analyses of the LAR–PSE relationship in the overall cohort.** | | | | | |
| --- | --- | --- | --- | --- | --- |
| Subgroup | Subgroup | Total (events of PSE) | OR (95% CI) | P Value | P Interaction* |
| DVT | No | 20135 (834) | 1.08 (0.99-1.17) | 0.072 | 0.227 |
|  | Yes | 1324 (102) | 1.41 (1.05-1.90) | 0.022 |  |
| Diabetes | No | 14137 (607) | 1.43 (1.22-1.67) | 0.000 | 0.000 |
|  | Yes | 7322 (329) | 1.00 (0.89-1.12) | 0.972 |  |
| Hypertension | No | 6708 (282) | 2.77 (1.89-4.05) | 0.000 | 0.000 |
|  | Yes | 14751 (654) | 1.02 (0.94-1.11) | 0.588 |  |
| Corory disease | No | 11787 (592) | 1.02 (0.91-1.13) | 0.751 | 0.874 |
|  | Yes | 9672 (344) | 1.25 (1.08-1.44) | 0.002 |  |
| Atrial fibrillation | No | 19416 (823) | 1.05 (0.96-1.15) | 0.255 | 0.126 |
|  | Yes | 2043 (113) | 1.47 (1.18-1.81) | 0.000 |  |
| Hydrocephalus | No | 21178 (878) | 1.12 (1.03-1.21) | 0.007 | 0.259 |
|  | Yes | 281 (58) | 9.94 (0.43-3034.81) | 0.215 |  |
| Hyperuricemia | No | 19112 (785) | 1.14 (1.05-1.23) | 0.002 | 0.001 |
|  | Yes | 2347 (151) | 1.82 (0.87-3.85) | 0.107 |  |
| Hyperlipidaemia | No | 16990 (786) | 1.09 (0.99-1.18) | 0.062 | 0.487 |
|  | Yes | 4469 (150) | 1.52 (1.19-1.94) | 0.001 |  |
| Hypoproteinemia | No | 19038 (760) | 1.07 (0.97-1.17) | 0.174 | 0.338 |
|  | Yes | 2421 (176) | 1.58 (1.19-2.12) | 0.002 |  |
| Frontal lobe | No | 20540 (853) | 1.10 (1.01-1.19) | 0.029 | 0.937 |
|  | Yes | 919 (83) | 1.42 (1.09-1.84) | 0.008 |  |
| Parietal lobe | No | 20796 (868) | 1.09 (1.00-1.19) | 0.039 | 0.270 |
|  | Yes | 663 (68) | 1.71 (1.28-2.30) | 0.000 |  |
| Temporal lobe | No | 20823 (871) | 1.07 (0.98-1.17) | 0.100 | 0.011 |
|  | Yes | 636 (65) | 2.08 (1.48-3.17) | 0.000 |  |
| Occipital lobe | No | 21064 (902) | 1.10 (1.01-1.19) | 0.021 | 0.100 |
|  | Yes | 395 (34) | 5.38 (1.95-20.88) | 0.004 |  |
| Insular lobe | No | 21180 (921) | 1.10 (1.02-1.19) | 0.018 | 0.078 |
|  | Yes | 279 (15) | 12.19 (3.20-205.91) | 0.010 |  |
| Basal ganglia | No | 20491 (878) | 1.07 (0.98-1.16) | 0.122 | 0.118 |
|  | Yes | 968 (58) | 2.23 (1.57-3.26) | 0.000 |  |
| Rhombencephalon | No | 21017 (918) | 1.12 (1.04-1.22) | 0.004 | 0.010 |
|  | Yes | 442 (18) | 0.31 (0.06-1.10) | 0.093 |  |
| Paraventricular | No | 20412 (882) | 1.06 (0.98-1.15) | 0.147 | 0.000 |
|  | Yes | 1047 (54) | 2.94 (1.92-4.57) | 0.000 |  |
| Thalamus | No | 21222 (919) | 1.11 (1.02-1.20) | 0.013 | 0.036 |
|  | Yes | 237 (17) | 1.12 (0.44-2.78) | 0.802 |  |
| MCA | No | 20631 (895) | 1.11 (1.02-1.21) | 0.011 | 0.871 |
|  | Yes | 828 (41) | 1.37 (1.00-1.94) | 0.064 |  |
| VA | No | 20807 (909) | 1.10 (1.01-1.19) | 0.021 | 0.141 |
|  | Yes | 652 (27) | 1.45 (0.82-2.71) | 0.210 |  |
| Gender | Male | 10616 (364) | 1.07 (0.96-1.19) | 0.232 | 0.353 |
|  | Female | 10843 (572) | 1.11 (0.97-1.26) | 0.124 |  |
| CCA plaque | No | 16636 (736) | 1.19 (1.09-1.30) | 0.000 | 0.013 |
|  | Yes | 4823 (200) | 1.04 (0.83-1.28) | 0.748 |  |
| ICA plaque | No | 19997 (860) | 1.14 (1.05-1.24) | 0.001 | 0.193 |
|  | Yes | 1462 (76) | 1.13 (0.81-1.56) | 0.457 |  |
| ECA plaque | No | 21258 (925) | 1.11 (1.02-1.20) | 0.009 | 0.035 |
|  | Yes | 201 (11) | 10.45 (1.74-135.57) | 0.030 |  |
| Subcortical areas | No | 19016 (801) | 0.99 (0.90-1.09) | 0.894 | 0.375 |
|  | Yes | 2443 (135) | 1.69 (1.40-2.04) | 0.000 |  |
| Anterior circulation | No | 20046 (868) | 1.10 (1.01-1.19) | 0.028 | 0.823 |
|  | Yes | 1413 (68) | 1.32 (1.03-1.72) | 0.034 |  |
| Posterior circulation | No | 16958 (762) | 1.07 (0.98-1.16) | 0.107 | 0.128 |
|  | Yes | 4501 (174) | 1.65 (1.31-2.07) | 0.000 |  |
| DVT, deep vein thrombosis; MCA, Middle cerebral artery; VA, Vertebral artery; CCA plaque; Common carotid artery plaque; ICA plaque; Internal carotid artery plaque; ECA plaque, External carotid artery plaque.  *All subgroup models were adjusted for the same set of covariates as in Model 3, excluding the stratification variable itself to prevent over-adjustment. | | | | | |

| **Table S3. Logistic regression analyses of the association between the LAR and PSE incidence in patients with acute ischemic stroke (age≤80 years).** | | | | | | | |
| --- | --- | --- | --- | --- | --- | --- | --- |
| Characteristics | Case/total, n | Model 1  OR (95%CI) | P value | Model 2  OR (95%CI) | P value | Model 3  OR (95%CI) | P value |
| LAR (per 1 mmol/g) | 827/18439 | 1.67 (1.59-1.76) | <0.001 | 1.80 (1.70-1.92) | <0.001 | 1.11 (1.02-1.21) | 0.02 |
| LAR quartile |  |  |  |  |  |  |  |
| Quartile 1 | 25/4617 | Reference |  | Reference |  | Reference |  |
| Quartile 2 | 53/4640 | 2.12 (1.33-3.48) | 0.002 | 3.11 (1.94-5.12) | <0.001 | 5.47 (3.09-10.13) | <0.001 |
| Quartile 3 | 141/4606 | 5.80 (3.86-9.10) | <0.001 | 9.49 (6.24-15.02) | <0.001 | 12.09 (7.12-21.70) | <0.001 |
| Quartile 4 | 608/4576 | 28.14 (19.26-43.19) | <0.001 | 45.18 (30.50-70.10) | <0.001 | 14.19 (8.48-25.05) | <0.001 |
| Model 1: unadjusted for any covariates.  Model 2: adjusted for age, gender, NIHSS, uremia, DVT, fatty liver, diabetes, hypertension, CAD, and atrial fibrillation.  Model 3: Model 2 + further adjusted for anterior circulation, posterior circulation, large vessel disease, PLT, WBC, RBC, HbA1c, TG, HDL-c, LDL-c, creatinine. | | | | | | | |

| **Table S4. Logistic regression analyses of the association between the LAR and PSE incidence in patients with acute ischemic stroke (without cerebral herniation patients).** | | | | | | | |
| --- | --- | --- | --- | --- | --- | --- | --- |
| **Characteristics** | **Case/total, n** | **Model 1**  **OR (95%CI)** | **P value** | **Model 2**  **OR (95%CI)** | **P value** | **Model 3**  **OR (95%CI)** | **P value** |
| LAR (per 1 mmol/g) | 916/21278 | 1.61 (1.54-1.69) | <0.001 | 1.73 (1.64-1.83) | <0.001 | 1.13 (1.05-1.22) | 0.002 |
| LAR quartile |  |  |  |  |  |  |  |
| Quartile 1 | 34/5338 | Reference |  | Reference |  | Reference |  |
| Quartile 2 | 58/5363 | 1.71 (1.12-2.63) | 0.014 | 2.41 (1.58-3.73) | <0.001 | 3.90 (2.42-6.44) | <0.001 |
| Quartile 3 | 177/5276 | 5.42 (3.80-7.96) | <0.001 | 8.47 (5.89-12.55) | <0.001 | 9.80 (6.39-15.55) | <0.001 |
| Quartile 4 | 647/5301 | 21.69 (15.58-31.27) | <0.001 | 34.12 (24.22-49.70) | <0.001 | 11.94 (7.85-18.79) | <0.001 |
| Model 1: unadjusted for any covariates.  Model 2: adjusted for age, gender, NIHSS, uremia, DVT, fatty liver, diabetes, hypertension, CAD, and atrial fibrillation.  Model 3: Model 2 + further adjusted for anterior circulation, posterior circulation, large vessel disease, PLT, WBC, RBC, HbA1c, TG, HDL-c, LDL-c, creatinine. | | | | | | | |

| **Table S5. Logistic regression analyses of the association between the LAR and PSE incidence in patients with acute ischemic stroke (without Fatty liver disease or steatosis).** | | | | | | | |
| --- | --- | --- | --- | --- | --- | --- | --- |
| **Characteristics** | **Case/total, n** | **Model 1**  **OR (95%CI)** | **P value** | **Model 2**  **OR (95%CI)** | **P value** | **Model 3**  **OR (95%CI)** | **P value** |
| LAR (per 1 mmol/g) | 795/17214 | 1.62 (1.54-1.71) | <0.001 | 1.77 (1.66-1.88) | <0.001 | 1.12 (1.03-1.22) | 0.01 |
| LAR quartile |  |  |  |  |  |  |  |
| Quartile 1 | 23/4345 | Reference |  | Reference |  | Reference |  |
| Quartile 2 | 49/4262 | 2.19 (1.35-3.66) | 0.002 | 3.24 (1.98-5.44) | <0.001 | 5.61 (3.18-10.29) | <0.001 |
| Quartile 3 | 155/4306 | 7.02 (4.62-11.18) | <0.001 | 11.54 (7.52-18.53) | <0.001 | 15.01 (8.97-26.54) | <0.001 |
| Quartile 4 | 568/4301 | 28.59 (19.27-44.74) | <0.001 | 49.03 (32.59-77.55) | <0.001 | 17.21 (10.33-30.31) | <0.001 |
| Model 1: unadjusted for any covariates.  Model 2: adjusted for age, gender, NIHSS, uremia, DVT, diabetes, hypertension, CAD, atrial fibrillation, and cerebral herniation.  Model 3: Model 2 + further adjusted for anterior circulation, posterior circulation, large vessel disease, PLT, WBC, RBC, HbA1c, TG, HDL-c, LDL-c, creatinine. | | | | | | | |

| **Table S6. Logistic regression analyses of the association between the LAR and PSE incidence in patients with acute ischemic stroke (without brainstem involvement).** | | | | | | | |
| --- | --- | --- | --- | --- | --- | --- | --- |
| **Characteristics** | **Case/total, n** | **Model 1**  **OR (95%CI)** | **P value** | **Model 2**  **OR (95%CI)** | **P value** | **Model 3**  **OR (95%CI)** | **P value** |
| LAR (per 1 mmol/g) | 920/21187 | 1.62 (1.55-1.70) | <0.001 | 1.74 (1.65-1.84) | <0.001 | 1.13 (1.04-1.22) | 0.003 |
| LAR quartile |  |  |  |  |  |  |  |
| Quartile 1 | 34/5312 | Reference |  | Reference |  | Reference |  |
| Quartile 2 | 57/5305 | 1.69 (1.11-2.61) | 0.016 | 2.42 (1.58-3.75) | <0.001 | 3.90 (2.41-6.45) | <0.001 |
| Quartile 3 | 175/5281 | 5.32 (3.73-7.82) | <0.001 | 8.48 (5.89-12.57) | <0.001 | 9.65 (6.28-15.33) | <0.001 |
| Quartile 4 | 654/5289 | 21.90 (15.74-31.58) | <0.001 | 35.24 (25.00-51.34) | <0.001 | 12.27 (8.05-19.36) | <0.001 |
| Model 1: unadjusted for any covariates.  Model 2: adjusted for age, gender, NIHSS, uremia, DVT, fatty liver, diabetes, hypertension, CAD, atrial fibrillation, and cerebral herniation.  Model 3: Model 2 + further adjusted for anterior circulation, posterior circulation, large vessel disease, PLT, WBC, RBC, HbA1c, TG, HDL-c, LDL-c, creatinine. | | | | | | | |
